# Analgesic Use in Patients with Mild Inflammatory Bowel Disease—A Nationwide Cohort Study Based on Prescription Data

**DOI:** 10.3390/jcm15083121

**Published:** 2026-04-20

**Authors:** Bente Mertz Nørgård, Caroline Theilgaard Thorarinsson, Kevin Erichsen Zeiss, Rahul S. Dalal, Mette Louise Andersen, Ken Lund, Sonia Friedman, Jens Kjeldsen, Torben Knudsen

**Affiliations:** 1 Center for Clinical Epidemiology, Odense University Hospital, 5000 Odense C, Denmark; 2 Research Unit of Clinical Epidemiology, Department of Clinical Research, University of Southern Denmark, 5000 Odense C, Denmark; 3 Division of Gastroenterology, Hepatology and Endoscopy, Department of Medicine, Brigham and Women’s Hospital, Harvard Medical School, Boston, MA 02130, USA; 4 Gastroenterology and Hepatology, Tufts Medical Centre, Boston, MA 02111, USA; 5 Department of Medical Gastroenterology S, Odense University Hospital, 5000 Odense C, Denmark; 6 Research Unit of Medical Gastroenterology, Department of Clinical Research, University of Southern Denmark, 5000 Odense C, Denmark; 7 Department of Medical Gastroenterology, Hospital of Southwest Jutland, 6700 Esbjerg, Denmark; 8 Department of Regional Health Science, University of Southern Denmark, 6700 Esbjerg, Denmark

**Keywords:** pain, analgesics, opioids, IBD, NSAID, paracetamol

## Abstract

**Background:** In patients with Crohn’s disease (CD) and ulcerative colitis (UC) disease severity and activity have been associated with pain. Data on analgesic use in patients with mild disease, however, are limited. We examined prescribed analgesics in mild CD and UC. **Methods:** In this cohort study, based on nationwide Danish prescription data, we identified incident patients (1996 through 2020) with mild CD (N = 5348) and UC (N = 15,622). Mild disease was defined by absence of advanced medical treatments, and no surgeries, in the period of 3.5 years after the diagnosis. We examined opioids, nonsteroidal anti-inflammatory drugs (NSAIDs), and paracetamol. We also examined opioid use in CD compared to UC. **Results:** In mild CD, the proportions of patients using analgesics in 2001 and 2020 were: strong/weak opioids 9.1% and 10.0%, chronic opioids 3.2% and 4.1%, NSAIDs 12.4% and 8.5%, and paracetamol 4.0% and 21.7%, respectively. In mild UC, the corresponding proportions in 2001 and 2020 were: strong/weak opioids 6.2% and 7.2%, chronic opioids 2.9% and 2.7%, NSAIDs 12.7% and 8.3%, and paracetamol 2.4% and 19.5%, respectively. The adjusted OR for chronic opioid use in mild CD relative to mild UC was 1.61 (95% CI 1.41–1.85). **Conclusions:** We found a widespread use of prescribed analgesics in patients with mild IBD. The trends across two decades were similar for both diseases: a steep increase in paracetamol, a modest decline in NSAIDs, a slight decline in weak opioids, but no decline in strong opioids. The risk of chronic opioid use was higher in mild CD than in mild UC. We suggest that patients with mild disease may have pain issues that need to be managed clinically.

## 1. Introduction

Inflammatory bowel diseases (IBD), including Crohn’s disease (CD) and ulcerative colitis (UC), are chronic inflammatory diseases of the gastrointestinal tract, and both diseases are highly heterogeneous in regard to activity and severity [1]. Former studies have shown a widespread use of analgesics in patients with IBD [2,3,4,5,6], but it remains unclear whether the use of analgesics is also linked to the subgroup of patients who have only mild disease after diagnosis. The perception of pain in patients with IBD is complex and may arise from multiple interacting mechanisms where both physiological and psychological factors have been suggested as contributing to the variability in pain experience [7].

At present, there is a lack of specific strategies for pain management in IBD or guidelines for subpopulations of patients at particular risk [8]. The traditional classes of analgesics utilized in IBD include opioids, nonsteroidal anti-inflammatory drugs (NSAIDs), and paracetamol (i.e., acetaminophen) [9,10]. There are several concerns related to analgesics. Opioids may mask the severity of IBD and delay medical care, which can lead to complications [11]. The use of NSAIDs has been associated with gastrointestinal toxicities [9], and chronic use of opioids is associated with addiction, severe infection, and mortality [7,12]. Despite these concerns, a large proportion of patients with IBD are prescribed analgesics. Wils et al. found that up to 60% experienced abdominal pain in their lifetime regardless of disease activity [7], and Targownik et al. found that within 10 years of diagnosis, 5% of patients with IBD became heavy opioid users [12]. A recent review suggests that the prevalence of prescription opioid use in the outpatient setting is 21% [2]. Also, our group has recently shown that approximately 25% of patients with IBD had chronic opioid use during 8 years of follow up [13].

Disease activity in IBD has been associated with pain and opioid use. Noureldin et al. showed that 35% of patients developed persistent opioid use after an IBD flare [5]. Patients with mild disease may also suffer from significant pain, but the data in this area are limited. In a study from the Netherlands, Janssen et al. showed that almost half of IBD patients in remission experienced chronic abdominal pain [14]. One of the challenges in this area is the substantial heterogeneity in the definition of mild IBD across European and American guidelines [15,16,17]. The term mild IBD often has various meanings depending on clinical context, i.e., whether it refers to the disease presentation at the time of diagnosis, symptoms that relate to activity at a particular time point, or the presence or absence of certain features observed during the diagnostic process [15,16,17].

It is unclear whether the use of analgesics is a significant problem in patients with mild IBD after the time of diagnosis. Therefore, based on nationwide Danish registry data, we aimed to describe the use of analgesics (opioids, NSAIDS, and paracetamol) in the years following the IBD-diagnosis in patients with mild IBD, defined using a newly developed algorithm [18]. We also examined whether trends in analgesic use have changed over the last decades and analyzed opioid use in patients with mild CD relative to those with mild UC.

## 2. Materials and Methods

### 2.1. Study Design and Setting

This is a nationwide cohort study based on the Danish health registries. In Denmark, the population constitutes approximately 6 million inhabitants where >90% are Caucasians, and all citizens have equal and free access to tax-supported health care services. It is mandatory by law to register all activities in the health care system into nationwide health registries. Therefore, access to data from these registries allowed us to conduct this study. All inhabitants have a unique civil registration number in the Civil Registration System, which provides the possibility for valid linkage of data between registries [19,20].

### 2.2. Data

We retrieved data from several registries. The Danish National Patient Registry includes data on all hospital contacts since 1977, including data on the date of admission and discharge, performed surgeries and procedures, and diagnoses according to the International Classification of Diseases (ICD). In this study, we used both version 8 (ICD-8) used prior to 1994 and version 10 (ICD-10) used from 1994 [21]. Additionally, surgeries are classified according to the Nordic Medico-Statistical Committee (NOMESCO) codes. All information on redeemed prescriptions has been collected in The Danish National Prescription Registry since 1994. Data in this registry include date of redemption and type of redeemed medications. All medications are classified according to the ATC (Anatomical Therapeutic Chemical Classification) system [22,23]. Lastly, we have used the Central Person Registration (CPR) System to obtain information on date of birth, sex, emigration, and mortal status.

### 2.3. Study Population of Patients with Mild IBD and Observation Period

We identified all adult (≥18 years old) patients with a first-time IBD-diagnosis between 1 January 1996 and 31 December 2020. This period allowed full follow up on all included patients. For a valid assessment of IBD, we required a minimum of two IBD-diagnoses registered in the National Patient Registry, and the patients were classified as having either CD (ICD-8 code: 56301 or ICD-10 code: K50) or UC (ICD-8 code: 56904 or ICD-10 code: K51) according to the latest given IBD-diagnosis [24]. From these, we selected the study population of patients with mild IBD. We used the first 6 months after the IBD-diagnosis as a washout period due to expected disease activity and allowed use of corticosteroids in this period (no use of biologics or IBD surgeries was allowed in the washout period). In a 3-year observation period, starting after the end of the washout period, we defined patients with mild IBD as patients who (i) did not receive prescriptions for corticosteroids, (ii) did not receive biologic treatments, and (iii) did not have IBD surgeries (Supplementary Figure S1 and Table S1) [25]. This definition of patients with mild IBD is in accordance with the requirements in a recently published paper from Jacobsen et al. [18]. All patients in the study cohort were thus observed for a total period of 3.5 years after the diagnosis (6 months washout period and 3 years thereafter). To avoid scenarios where the use of analgesics was related to cancer, we excluded all patients who had a cancer diagnosis prior to the IBD-diagnosis, or during the observation period. Patients with other conditions that could theoretically be linked to pain were not excluded, as such conditions are difficult to define, and the vast majority of patients with IBD had no comorbidities.

### 2.4. Outcomes

The outcome of interest was use of analgesics in a three-year period after the first IBD-diagnosis. The types of analgesics included NSAIDs, paracetamol (i.e., acetaminophen) and opioids (weak and strong; Supplementary Table S2 for codes). Weak and strong opioids were divided into categories (0, 1, 2–5, ≥6) based on the number of filled prescriptions during the observation period. Additionally, we examined ≥ 1 prescription of either weak or strong opioids as a combined (yes/no) outcome, and chronic opioid use (yes/no). Chronic opioid use was defined as at least three opioid prescriptions within a 12-month period starting from the date of the first opioid prescription and with at least 30 days between each opioid prescription (Supplementary Figure S2) [26,27]. This algorithm for chronic opioid use has been found robust across modified algorithms [26]. We also examined the use of NSAIDs and paracetamol as separate outcomes (yes/no), as well as use of either NSAIDs or paracetamol as a combined outcome (yes/no).

### 2.5. Covariates

Age at first IBD-diagnosis was divided into two age-groups: ‘young adults’ (18–39 years) and ‘adults/elderly’ (40+ years) [28]. Calendar year of first IBD-diagnosis was divided into periods (1996–2000, 2001–2005, 2006–2010, 2011–2015, and 2016–2020) for descriptive purposes. Use of methotrexate (yes/no), thiopurines (yes/no) or 5-ASA (yes/no) during follow up was based on information from the National Prescription Registry and the National Patient Registry (Supplementary Table S3 for IBD medication codes). Sex at birth (female/male) was retrieved from the CPR system. Charlson Comorbidity Index (CCI) was calculated within a 10-year period before the first IBD-diagnosis and was divided into the categories of low (CCI = 0), medium (CCI = 1 or CCI = 2), and high comorbidity (CCI > 2). Diseases other than those included in CCI may be linked to pain conditions in IBD, and therefore we also retrieved information on underlying rheumatic diseases (ICD-10 codes: M02, M05, M06, M070-M073, M08, M10, M11, M3, M45, M46 and L40), irritable bowel syndrome (ICD-10 codes: K58, K580 and K589), and mental illnesses/behavioral disorders (ICD-10 codes: F00–F99). These underlying diseases (rheumatic diseases (yes/no), irritable bowel syndrome (yes/no), mental illnesses/behavioral disorders (yes/no)) were examined within a period of two years prior to the first IBD-diagnosis until the end of the observation period. The period of two years prior to the IBD-diagnosis was chosen as we only found it relevant to consider the most recent diseases in these categories (and not, for example, a diagnosis of irritable bowel syndrome that was recorded many years prior to IBD-diagnosis).

### 2.6. Statistical Analysis

We constructed contingency tables for the main study variables according to patients with CD and UC. For patients with UC and CD, we calculated the proportion of patients who used different types of analgesics according to calendar year and age. The proportion in each year was calculated according to the following: the numerator was the number of patients who met the criteria for analgesic use in a given calendar year, and the denominator was the total number of patients who contributed with observation time in the same year. These proportions were used in all graphs where we visualized the trends in the proportions of filled prescriptions of paracetamol, NSAIDs, strong and weak opioids, and chronic opioid use by calendar years. Non-parametric trend test (Jonckheere–Terpstra) was applied to all figures to assess monotonic changes in proportions across the calendar years. In graphs we stratified the analyses by types of analgesics and age groups. The exact proportions with 95% CIs underlying the graphs are presented in Supplementary Table S4.

In logistic regression models we calculated crude and adjusted odds ratios (OR) with 95% confidence intervals (95% CI) to estimate whether patients with mild CD relative to patients with mild UC were at higher risk to be prescribed opioids (weak or strong) or develop chronic opioid use. In the model, we adjusted for variables that might be linked to opioid use, i.e., calendar year (as a continuous variable), age (categorized as young adults and adults/elderly), and the presence of underlying diseases (rheumatic disease, irritable bowel syndrome, or mental illnesses/behavioral disorders, each coded as yes/no). The use of thiopurines and methotrexate was different between CD and UC. Therefore, we also adjusted for these during the three-year follow-up period, each coded as yes/no. In a sub-analysis, we excluded all women from the study population who gave birth or had pregnancies ending in abortions during the observation period. This was done to ensure that our results were not confounded by analgesics used for pain-related conditions in pregnancy.

In another sub-analysis, we examined prescribed opioids (weak or strong) stratified by sex. We tried to stratify our analyses according to co-existing rheumatic diseases, but these were underpowered. The same issue of limited data applied when we attempted to stratify the population into additional age categories. In a sensitivity analysis we reduced the washout period from 6 to 4 months.

## 3. Results

We included 5348 patients with mild CD and 15,622 patients with mild UC. Descriptive characteristics are provided in Table 1. Most patients had CD and UC onset from 18 to 39 years of age (61.4% and 51.1%, respectively). We found increasing proportions of patients with mild disease when examined by calendar periods (CD: 17.5% in 2006–2010 to 30.0% in 2016–2020. UC: 19.9% in 2006–2010 to 26.2% in 2016–2020). The majority of patients with IBD had a low CCI, and rheumatic diseases were identified in 4.6% of patients with CD and 2.8% of patients with UC.

### 3.1. Overall Analgesic Use in CD and UC Patients with Mild Disease

In CD, 25.4% (95% CI 24.2–26.5%) of patients were prescribed weak or strong opioids across the included calendar years. The corresponding proportion in UC was 19.3% (95% CI 18.6–19.8%). In CD, 42.8% (95% CI 41.4–44.1%) of patients were prescribed NSAIDs or paracetamol across the included calendar years. The corresponding proportion in UC was 38.4% (95% CI 37.6–39.2%).

### 3.2. Use of Analgesics by Calendar Period in Mild CD

**Figure 1:**
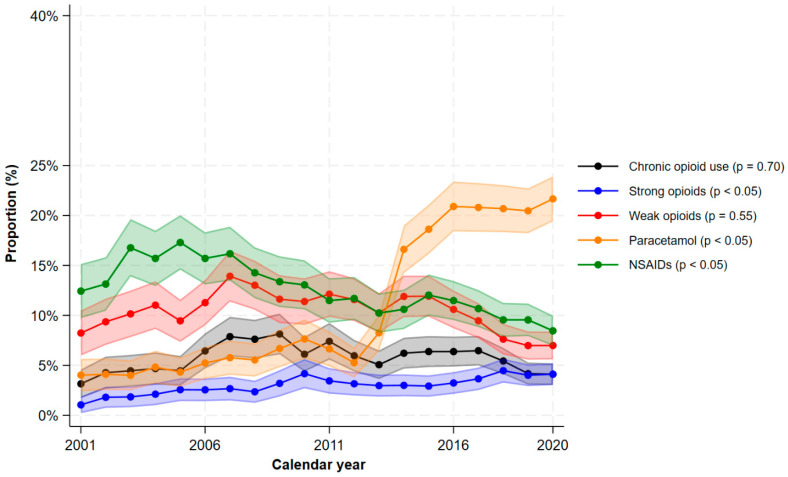
The proportions of filled prescriptions of analgesics among patients with Crohn’s disease by calendar period. The shaded areas correspond to the 95% confidence interval. *p*-values < 0.05 indicates a significant monotonic trend.

**Figure 2:**
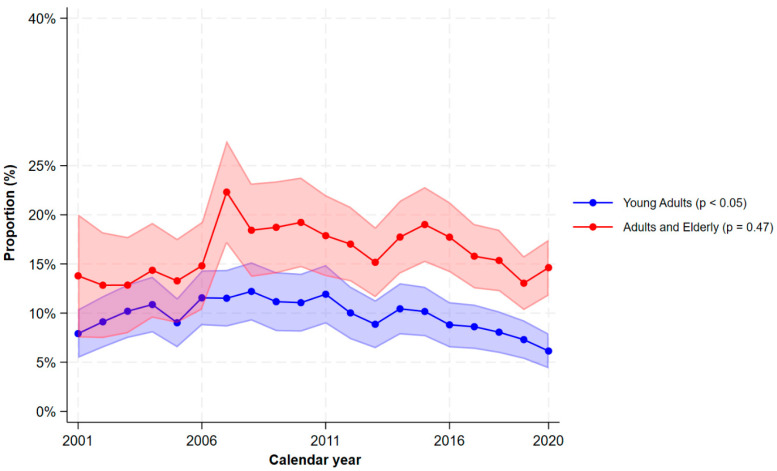
The proportions of filled prescriptions of opioids (weak or strong) among patients with Crohn’s disease by calendar period and stratified by age groups. The shaded areas correspond to the 95% confidence interval. *p*-values < 0.05 indicates a significant monotonic trend.

A large proportion of patients with mild CD used analgesics (Figure 1). In 2020, the proportions using paracetamol were 21.7% (95% CI 19.4–23.9%), NSAIDs 8.5% (95% CI 6.9–10.0%), weak opioids 7.0% (95% CI 5.6–8.4%), strong opioids 4.1% (95% CI 3.0–5.2%), weak or strong opioids 10.0% (8.4–11.7%), and chronic opioids 4.1% (3.0–5.2%). The use of paracetamol increased dramatically since 2013 (Figure 1), but during the last decade, the proportion of patients using strong opioids did not decline (4.1% in 2020). Figure 2 shows the stratified analyses according to age groups at first IBD-diagnosis. In all calendar years, the overall use of opioids was more prevalent among adults/elderly compared to young adults, and in 2020, 14.6% (95% CI 11.8–17.5%) of adults/elderly used opioids versus 6.1% (95% CI 4.4–7.9%) of young adults.

### 3.3. Use of Analgesics by Calendar Period in Mild UC

**Figure 3:**
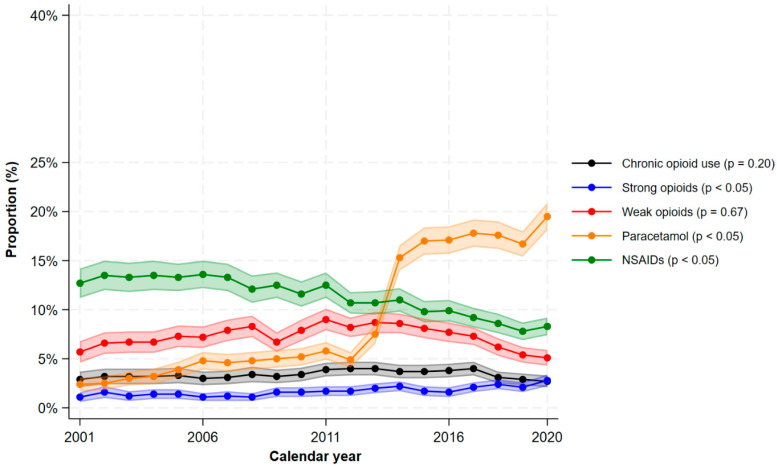
The proportions of filled prescriptions of analgesics among patients with ulcerative colitis by calendar period. The shaded areas correspond to the 95% confidence interval. *p*-values < 0.05 indicates a significant monotonic trend.

**Figure 4:**
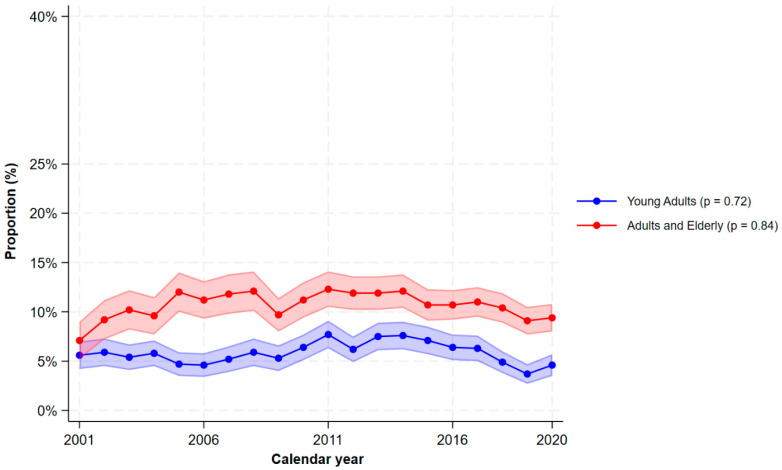
The proportions of filled prescriptions of opioids (weak or strong) among patients with ulcerative colitis by calendar time and stratified by age groups. The shaded areas correspond to the 95% confidence interval. *p*-values < 0.05 indicates a significant monotonic trend.

A large proportion of patients with mild UC also used analgesics (Figure 3). In 2020, the proportions using paracetamol were 19.5% (95% CI 18.1–20.9%), NSAIDs 8.3% (95% CI 7.4–9.2%), weak opioids 5.1% (95% CI 4.3–5.9), strong opioids 2.8% (95% CI 2.2–3.4%), weak or strong opioids 7.2% (95% CI 6.3–8.1%), and chronic opioids 2.7% (95% CI 2.1–3.3). Similarly to CD, the use of paracetamol increased dramatically. During the last decade, the proportion of patients using weak opioids declined (9.0% (95% CI 7.9–10.1%) in 2011 to 5.1% (95% CI 4.3–5.9%) in 2020). Chronic opioid use also declined (3.9% (95% CI 3.2–4.6%) in 2011 to 2.7% (95% CI 2.1–3.3%) in 2020). However, the use of strong opioids increased (1.7% (95% CI 1.2–2.2%) in 2011 to 2.8% (95% CI 2.2–3.4%) in 2020). Figure 4 shows the stratification by age groups. The overall use of opioids was more prevalent among adults/elderly compared to young adults, and in 2020, 9.4% (95% CI 8.0–10.8%) of the adults/elderly used opioids versus 4.6% (95% CI 3.5–5.7%) of young adults.

### 3.4. Opioids in Mild CD Versus Mild UC

Table 2 shows the crude and adjusted ORs for opioids in patients with mild CD, relative to patients with mild UC. In mild CD, the adjusted OR for use of opioids (weak or strong) was 1.39 (95% CI 1.28–1.50), and for chronic opioid use, it was 1.61 (95% CI 1.41–1.85).

### 3.5. Sub-Analysis and Sensitivity Analysis

The sub-analysis excluding women who gave birth or had pregnancies ending in abortions did not change the conclusions from the primary analysis (adjusted OR for weak or strong opioids 1.35 (95% CI 1.23–1.46) and the adjusted OR for chronic opioid use 1.51 (95% CI 1.31–1.74)).

Our conclusions also did not change in the sensitivity analyses where we restricted the washout period from 6 to 4 months (adjusted OR for weak or strong opioids 1.39 (95% CI 1.28–1.51) and the adjusted OR for chronic opioid use 1.61 (95% CI 1.40–1.85)).

In sub-analyses we visualized prescribed opioids (weak or strong) stratified by sex (Supplementary Figures S3 and S4).

## 4. Discussion

This nationwide cohort study of patients with mild IBD demonstrated widespread use of prescribed opioids and non-opioid analgesics in the years following IBD-diagnosis. We also showed that the risk of chronic opioid use was higher in mild CD compared to mild UC. During the last two decades, we found that the trends for analgesic use in patients with mild CD and UC have been the same: a dramatic increase in prescriptions of paracetamol, a modest decline in prescriptions of NSAIDs, and a slight decline in prescriptions of weak opioids. We did not find a decline in prescriptions of strong opioids from 2001 to 2020, and the proportion of patients with chronic opioid use remained largely stable over the past two decades. Unfortunately, our data cannot determine the underlying reasons behind these figures. An increased awareness against prescription of opioids has been an important topic in Denmark, and we might see an effect of these initiatives in the future.

To our knowledge, this study presents real-word data on analgesic use on the largest number of patients with mild IBD. We found both an increasing number and proportion of patients with mild disease by calendar period in CD and UC. This is most likely a reflection of the general increasing incidence of IBD. Many concerns are related to the use of opioids, and one might speculate whether the use of opioids in the population of patients with mild IBD is in fact higher than in the background Danish population. Our best estimates for overall use in the background population are between 2 and 6% depending on the way it is analyzed. A recent Danish study found 1,683,713 unique opioid users in a cancer free population of individuals in a 14-year study period, corresponding to an average of 120,262 new users each year, and a proportion of approximately 2% [29]. According to MEDSTAT (Danish health authority’s surveillance of medication use) the most recent proportion of overall use of opioids is closer to 6%, but this proportion is not comparable to ours, as our population excluded cancer patients [30]. Therefore, such data indicate that our findings regarding the use of weak/strong opioids in patients with mild CD (10.0%) is higher than in the background population, and also higher in patients with UC (7.2%). We found a dramatic increase in prescribed paracetamol since 2013 and this corresponds to a change in the access to paracetamol. Due to policy changes to reduce the risk of poisoning and suicide attempts, large packages could no longer be sold over the counter, and therefore patients needing long-term use of paracetamol required prescriptions. This regulation is most likely the explanation for the increase since 2013. The finding of higher overall opioid use among adults and the elderly compared to young adults is consistent with other studies [4,13,31].

Our results cannot be directly compared with those of other studies, as mild or quiescent IBD has been defined differently. Terms such as “mild disease,” “inactive disease,” “quiescent disease”, or “disease in remission” vary between studies and are based on the specific information available, such as medication use, disease activity scores, endoscopic or biochemical remission, among others. A recent review from 2025 highlights the substantial proportion of patients with mild IBD and emphasizes that a considerable proportion of patients with UC achieve remission on 5-ASA without requiring advanced therapies or surgery; however, the definition of mild CD remains insufficiently standardized [32]. Despite these challenges in defining mild IBD, attempts have been made to describe pain in this patient group. In a study from the Netherlands, Janssen et al. examined 429 patients in biochemical remission and examined patient characteristics in those with and without abdominal pain [14]. Data were based on questionnaires during an 18-month period. The presence of abdominal pain was based on self-reported pain scores, and the use of analgesics was not assessed. They found that 46.2% of the patients in remission experienced chronic abdominal pain, which was associated with problems related to sex, fatigue, and depression over time [14].

A general problem in the area of IBD and pain is that there is currently no consensus definition of abdominal pain or chronic analgesic use in the medical literature. Former studies of IBD patients in remission have reported pain prevalence ranging from 12% to 50%, but they included variable definitions of abdominal pain based on pain scores and/or use of analgesics [14,33,34,35]. To our knowledge, use of analgesics in large cohorts of patients specifically with mild IBD has not been reported previously. The perception of abdominal pain by IBD patients is complex and has been explained by different mechanisms with multiple contributing factors [7]. Wils et al. outlined a pain model of contributing factors including (i) direct effect of inflammation, (ii) peripheral and central pain dysregulation involving the enteric nervous system, the central nervous system, the gastro-intestinal immune system, and the intestinal barrier, (iii) overlap to irritable bowel syndrome, (iv) impact of psychological and social factors on pain perception, and (v) genetic predisposition to pain [7].

Prior studies have estimated pain symptoms related to irritable bowel syndrome in patients with mild disease [33,35,36]. In a systematic review and meta-analysis, Faribrass et al. examined the prevalence of irritable bowel syndrome symptoms, characterized by features of abdominal pain, and found that these symptoms varied according to how remission was defined [33]. However, even after adopting more stringent criteria such as endoscopic or histological remission, about a quarter of patients reported pain symptoms. In a questionnaire study, Farrokhyar et al. examined irritable bowel syndrome symptoms in 149 patients who had quiescent IBD (defined by no additions or changes in therapy) for a period of 12 months, and 12% reported abdominal pain [35]. A survey study of 334 patients by Schirbel et al. found that 12.1% of patients reported no pain and 39.7% only had pain during flare-ups, while 48.2% reported persistent pain [34]. Other studies support our finding that pain is more common in quiescent CD than in quiescent UC. Farrokhyar at al. found that irritable bowel syndrome symptoms were more common in inactive CD than in UC (26% versus 9.1%, *p* = 0.01) [35], and a similar finding was shown by Faribrass et al. [33].

This study has several strengths. It is a major strength that we had access to mandatory and routinely collected nationwide data, which increases the generalizability of our results. Danish heath registries provide valid data, which are important tools for clinical epidemiological studies [20,21,23,37]. We have complete follow up on all included patients with IBD, which prevents selection bias. It is a strength that our algorithm for identification of patients with mild IBD is consistent with other published data [18]. However, a potential misclassification of mild IBD cannot be ruled out. We had complete analgesic prescription data for all patients, which is an important advantage over survey studies that may suffer from recall bias. We chose to investigate the use of analgesics over a period of 3.5 years following the diagnosis of IBD. We considered this to be a reasonable timeframe, as extending the observation period further would likely increase the risk that analgesics were prescribed for non-IBD indications.

The limitations of the study are linked to the use of administrative data. Thus, progression related variables, such as extent of disease, stricturing/penetrating disease, endoscopic inflammation, and biological markers were either unavailable or lacked sufficient quality. We do not believe we have overestimated the magnitude of prescribed analgesics. On the contrary, we have likely underestimated analgesic use, as over-the-counter use of small packages of paracetamol and weak NSAIDs are not recorded in the prescription registry. The use of over-the-counter NSAIDs in patients with IBD is unknown. The Danish National Prescription Registry also does not include the medical indications of prescriptions and does not include information regarding the intended duration or dosage of medications [23]. We are therefore unable to confirm if analgesics were prescribed specifically for IBD or other conditions, though we have ensured that analgesics are not prescribed for cancer-related pain. Furthermore, it would have been valuable to include a comparison group from the general population, although it would be difficult to define how such an appropriate sample should be constructed. Even though the registry data do not include detailed information on clinical parameters and disease activity scoring systems, an observational study like this is important despite its inherent limitations. As with other observational studies, it is not possible to exclude an influence of unknown confounders or residual confounding. Regarding generalizability, our results may apply to countries with health care systems similar to Denmark.

## 5. Conclusions

In patients with mild IBD, our study reveals widespread use of prescribed opioids as well as non-opioid analgesics, and we demonstrate that opioids are more often prescribed in patients with mild CD than in mild UC. Given the large proportion of patients with mild IBD, who are prescribed analgesics, further investigations are needed in several areas. First, a consensus definition of mild IBD is needed. Future research should also attempt to characterize patient reported use of over-the-counter analgesics and the specific reasons and symptoms prompting analgesic use in general. To reduce opioid use and their associated complications, future studies should attempt to identify and test alternative pain management strategies that can be implemented into clinical guidelines.

## Figures and Tables

**Table 1:** Descriptive table of characteristics of patients with Crohn’s disease and ulcerative colitis with mild disease.

	Crohn’s Disease N = 5348	Ulcerative Colitis N = 15,622
Age at first diagnosis, N (%)		
Young (18–39 years)	3281 (61.4)	7977 (51.1)
Adults and Elderly (40+ years)	2067 (38.6)	7645 (48.9)
Year of first diagnosis, N (%)		
1996–2000	717 (13.4)	2137 (13.7)
2001–2005	895 (16.7)	2704 (17.3)
2006–2010	934 (17.5)	3107 (19.9)
2011–2015	1198 (22.4)	3582 (22.9)
2016–2020	1604 (30.0)	4092 (26.2)
Sex, N (%)		
Female	3019 (56.5)	8402 (53.8)
Male	2329 (43.5)	7220 (46.2)
IBD-medication used in the observation period (≥1 prescription) *, N (%)		
Thiopurines	1241 (23.2)	609 (3.9)
MTX	89 (1.7)	96 (0.6)
5-ASA	1340 (25.1)	11,496 (73.6)
Analgesics used in observation period * (≥1 prescription), N (%)		
NSAIDs or paracetamol	2290 (42.8)	6002 (38.4)
NSAIDs	1640 (30.7)	4449 (28.5)
Paracetamol	1310 (24.5)	3192 (20.4)
Weak or strong opioids	1358 (25.4)	3008 (19.3)
Chronic opioid use	397 (7.4)	747 (4.8)
Number of prescriptions of weak opioids filled in observation period, N (%)		
0	4173 (78.0)	12,953 (82.9)
1	553 (10.3)	1410 (9.0)
2–5	373 (7.0)	764 (4.9)
≥6	249 (4.7)	495 (3.2)
Number of prescriptions of strong opioids filled in observation period, N (%)		
0	4946 (92.5)	14,907 (95.4)
1	164 (3.1)	318 (2.0)
2–5	126 (2.4)	209 (1.3)
≥6	112 (2.1)	188 (1.2)
Underlying diseases, N (%)		
Rheumatic disease	245 (4.6)	441 (2.8)
Irritable bowel syndrome	501 (9.4)	756 (4.8)
Mental illnesses/behavioral disorders	405 (7.6)	714 (4.6)
CCI **, N (%)		
Low comorbidity (CCI = 0)	4747 (88.8)	14,195 (90.9)
Medium comorbidity (CCI = 1–2)	558 (10.4)	1323 (8.5)
High comorbidity (CCI > 2)	43 (0.8)	104 (0.7)

* In the three-year period after the first IBD-diagnosis (calculated from the end of wash-out period) ** CCI = Charlson Comorbidity Index (calculated within a 10 year period before the first IBD-diagnosis).

**Table 2:** Crude and adjusted ORs (with 95% CI) for use of opioids (weak or strong), and chronic opioid use, during the observation period in patients with Crohn’s disease, relative to patients with ulcerative colitis.

		Crohn’s Disease, N = 5348	Ulcerative Colitis, (Reference) N = 15,622	Crude OR (95% CI)	Adjusted OR (95% CI) ^a^
At least 1 prescription of opioids in the three year observation period					
	Yes, N (%)	1358 (25.39)	3008 (19.25)	1.43 (1.33–1.55)	1.39 (1.28–1.50)
	No, N (%)	3990 (74.61)	12,614 (80.75)	-	-
Chronic opioid use ^b^ in the three year observation period					
	Yes, N (%)	397 (7.42)	747 (4.78)	1.60 (1.40–1.81)	1.61 (1.41–1.85)
	No, N (%)	4951 (92.58)	14,875 (95.22)	-	-

a: adjusted for calendar year (continuously), age group, gender, presence of underlying diseases according to diagnoses of rheumatic disease, irritable bowel syndrome or mental illnesses/behavioral disorders, use of thiopurines, and use of methotrexate during the three year follow-up period. b: Chronic opioid use was defined as at least three opioid prescriptions within a 12-month period starting from the date of the first opioid prescription and with at least 30 days between each opioid prescription.

## Data Availability

We are not allowed to share patient data with others. We do not have special access privileges to the data used in this study. Other researchers can have access to the Danish registry data through an application to the Danish Data Authority (forskerservice@sundhedsdata.dk). Access to data also requires approval from the Danish Data Protection Agency.

## Supplementary Materials

The following supporting information can be downloaded at: https://www.mdpi.com/article/10.3390/jcm15083121/s1, Figure S1: Incident patients with IBD (≥18 years) with mild disease. Figure S2: Illustration of chronic opioid use. Figure S3: The proportions of filled prescriptions of opioids (weak or strong) among patients with Crohn’s disease by calendar period and stratified by sex. Figure S4: The proportions of filled prescriptions of opioids (weak or strong) among patients with ulcerative colitis by calendar period and stratified by sex. Table S1: Surgery codes. Table S2: Codes for analgesics. Table S3: IBD medication codes. Table S4: Exact proportions with 95% CI for Figure 1 and Figure 3.

## Patient and Relative-Involvement

Patient and relative representatives are part of the research council at the Center for Clinical Epidemiology, Odense University Hospital. They have participated in the discussion of the purpose of this study on analgesic use in IBD. Patient and public representatives have not been part of the design of the study, data analyses, nor in the writing of the manuscript.
