# Supplementary material for: Analgesic Use in Patients with Mild Inflammatory Bowel Disease—A Nationwide Cohort Study Based on Prescription Data
Source: J Clin Med. 2026 Apr 20;15(8):3121. doi: 10.3390/jcm15083121 (PMC13117157; doi:10.3390/jcm15083121)

## Supplementary figure and tables

**Figure S1:** Incident patients with IBD (≥18 years) with mild

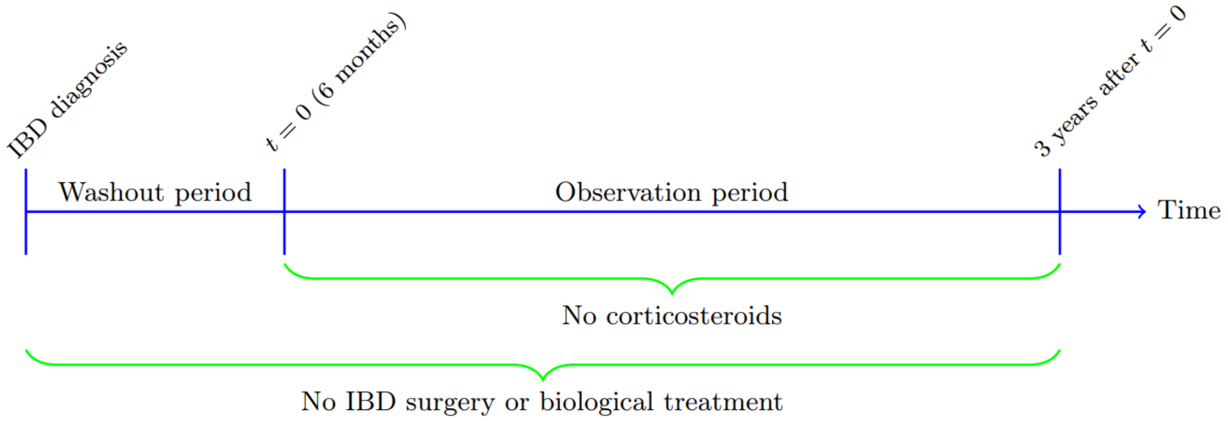


**Figure S2**: Illustration of chronic opioid use.

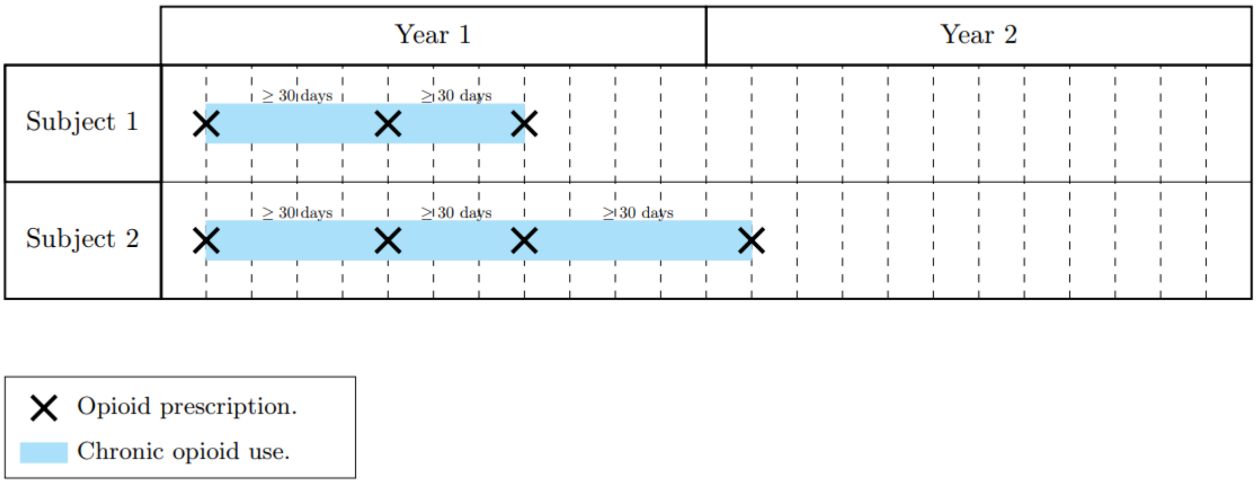


**Supplementary Table S1**: Surgery codes

| | **ICD-10 codes** |
|---|---|
| CD surgeries | |
| Ileocecal resection | KJFB20, KJFB21 |
| Ileocolonic resection | KJFB30, KJFB031, KJFB33, KJFB34, KJFH00, KJFH01 |
| Small bowel resection or stricturoplasty | KJFB00, KJFB01, KJFA60 |
| Stomas but rectum left in place | KJFH10, KJFH11, KJFB60 |
| Colon Resection | KJFB40, KJFB41, KJFB43, KJFB44, KJFB46, KJFB47, KJFB50, KJFB51, KJFB61, KJFB63, KJFB64, KJFH96 |
| Rectal/anus resection | KJGB00, KJGB01, KJGB10, KJGB11, KJGB30, KJGB31, KJFH20. |

| | |
|---|---|
| Other bowel resections | KJFB96, KJFB97 |
| UC surgeries<br>Colectomies | <br>KJFH |

**Supplementary Table S2**: Codes for analgesics

| **Medication classes** | **ATC codes** |
|---|---|
| **Opioids** | |
| Morphine (s) | N02AA01 |
| Hydromorphone (s) | N02AA03 |
| Nicomorphine (s) | N02AA04 |
| oxycodone (s) | N02AA05 |
| oxycodone and naloxone combination products (s) | N02AA55 |
| Pethidine (s) | N02AB02 |
| fentanyl (s) | N02AB03 |
| Buprenorphine (s) | N02AE01 |
| ketobemidone and antispasmodic combination products (s) | N02AG02 |
| Tapentadol (s) | N02AX06 |
| Methadone (s) | N07BC02 |
| codeine and paracetamol combination products (w) | N02AJ06 |
| codeine and acetylsalicylic acid combination products (w) | N02AJ07 |
| codeine (w) | R05DA04 |
| tramadol (w) | N02AX02 |
| Dextropropoxyphene (w) | N02AC04 |
| **NSAIDs** | M01A (except M01AX05) |
| **Paracetamol** | N02BE |

W= weak opioid. S= strong opioid

**Supplementary Table S3.** IBD medication

| codes | **ATC codes** | **Treatment codes** |
|---|---|---|
| Thiopurines | L04AX01 and L01BB02 | BWHB83 |
| Biological treatment (infliximab, adalimumab, golimumab, ustekinumab and vedolizumab) | L04AB (but not L04AB01 and L04AB05), L04AC05 and L04AA33 | BOHJ18A1, BOHJ18A3, BOHJ18A4, BOHJ18B3 and BOHJ19H4 |
| 5-ASA | A07EC | - |
| Systemic corticosteroids | H02AB01, H02AB02, H02AB04, H02AB06, H02AB07 and H02AB09 | - |
| Methotrexate | L04AX03 and L01BA01 | - |

**Supplementary Table S4**. Exact proportions with 95% CI for Figures 1 and 3.

Percentages for Figure 1 (analgesics in CD)

| CD | | | | | |
|---|---|---|---|---|---|
| **Year** | 2001 (95% CI) | 2006 (95% CI) | 2011 (95% CI) | 2016 (95% CI) | 2020 (95% CI) |
| **Strong or weak opioids** | 9.1 (6.7-11.5) | 12.6 (10.2-15.0) | 14.4 (12.0-16.9) | 12.7 (10.6-14.7) | 10.0 (8.4-11.7) |
| **Strong opioids** | 1.1 (0.2-2.0) | 2.6 (1.4-3.7) | 3.4 (2.2-4.7) | 3.2 (2.2-4.3) | 4.1 (3.0-5.2) |
| **Weak opioids** | 8.2 (6.0-10.5) | 11.3 (9.0-13.5) | 12.1 (9.8-14.4) | 10.6 (8.7-12.5) | 7.0 (5.6-8.4) |
| **NSAIDs** | 12.4 (9.7-15.1) | 15.7 (13.1-18.3) | 11.5 (9.3-13.7) | 11.5 (9.5-13.4) | 8.5 (6.9-10.0) |
| **Paracetamol** | 4.0 (2.4-5.6) | 5.2 (3.6-6.8) | 6.6 (4.9-8.4) | 20.9 (18.4-23.4) | 21.7 (19.4-23.9) |
| **Chronic opioid use** | 3.2 (1.7-4.6) | 6.4 (4.7-8.2) | 7.4 (5.6-9.2) | 6.4 (4.9-7.9) | 4.1 (3.0-5.2) |

Percentages for Figure 3 (analgesics in UC)

| UC | | | | | |
|---|---|---|---|---|---|
| **Year** | 2001 (95% CI) | 2006 (95% CI) | 2011 (95% CI) | 2016 (95% CI) | 2020 (95% CI) |
| **Strong or weak opioids** | 6.2 (5.1-7.3) | 7.7 (6.6-8.8) | 9.9 (8.8-11.0) | 8.6 (7.6-9.6) | 7.2 (6.3-8.1) |
| **Strong opioids** | 1.1 (0.6-1.6) | 1.1(0.7-1.5) | 1.7 (1.2-2.2) | 1.6 (1.1-2.1) | 2.8 (2.2-3.4) |
| **Weak opioids** | 5.7 (4.6-6.8) | 7.2 (6.1-8.3) | 9.0 (7.9-10.1) | 7.7 (6.7-8.7) | 5.1 (4.3-5.9) |
| **NSAIDs** | 12.7 (11.2-14.2) | 13.6 (12.2-15.0) | 12.5 (11.2-13.8) | 9.9 (8.8-11.0) | 8.3 (7.4-9.2) |
| **Paracetamol** | 2.4 (1.7-3.1) | 4.8 (3.9-5.7) | 5.8 (4.9-6.7) | 17.1 (15.7-18.5) | 19.5 (18.1-20.9) |
| **Chronic opioid use** | 2.9 (2.1-3.7) | 3.0 (2.3-3.7) | 3.9 (3.2-4.6) | 3.8 (3.1-4.5) | 2.7 (2.1-3.3) |

**Figure S3** The proportions of filled prescriptions of opioids (weak or strong) among patients with Crohn's disease by calendar period and stratified by sex. The shaded areas correspond to the 95% confidence interval. **P-values < 0.05 indicates a significant monotonic trend.**

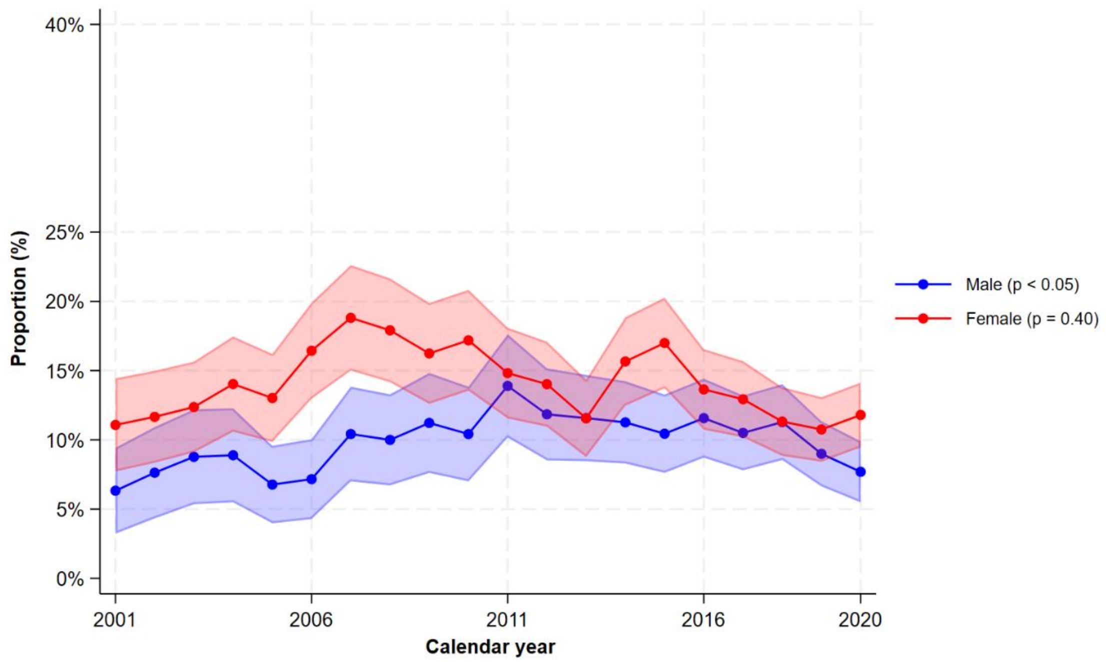

40%
25%
20%
15%
10%
5%
0%
Proportion (%)
2001
2006
2011
2016
2020
Calendar year
Male (p < 0.05)
Female (p = 0.40)

**Figure S4** The proportions of filled prescriptions of opioids (weak or strong) among patients with ulcerative colitis by calendar period and stratified by sex. The shaded areas correspond to the 95% confidence interval. **P-values < 0.05 indicates a significant monotonic trend.**

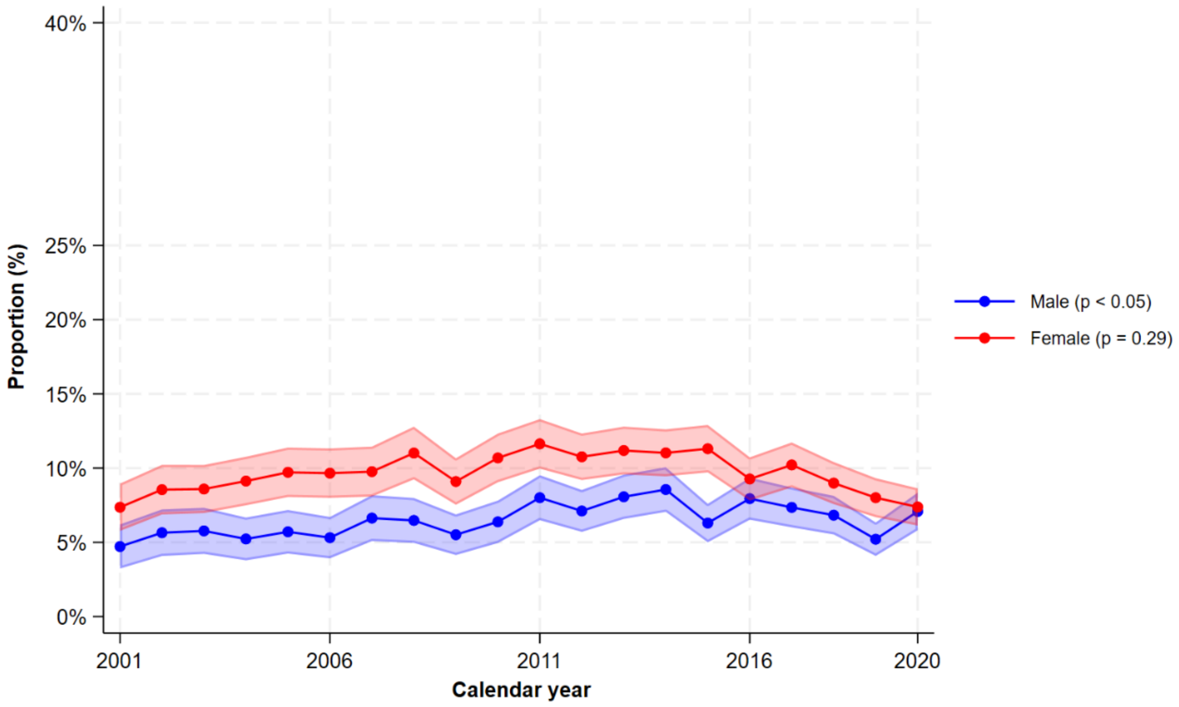

Supplement: Supplementary file 1 [file jcm-15-03121-s001.zip › jcm-4215360-supplementary.pdf]
